# Bias and discrimination perceived by antimicrobial stewards: a mixed-methods study

**DOI:** 10.1017/ice.2025.10224

**Published:** 2025-09

**Authors:** Jessica Tischendorf, Allison Giuffre, Katherine Cinnamon, Michael Howe, Fauzia Hollnagel, Lindsay Taylor

**Affiliations:** 1 Department of Medicine, Division of Infectious Disease, University of Wisconsin School of Medicine and Public Health, Madison, WI, USA; 2 Department of Medicine, University of Wisconsin School of Medicine and Public Health, Madison, WI, USA; 3 Department of Pharmacy, William S. Middleton Memorial Veterans Hospital, Madison, WI, USA; 4 Oak Ridge Institute for Science and Education, Oak Ridge, TN, USA

## Abstract

**Background:**

Bias and discrimination influence the experience of many in health care, including antimicrobial stewardship providers. In this mixed-methods study, we explore the perceptions of bias and discrimination among antimicrobial stewards.

**Methods:**

We conducted a nationwide survey of stewardship providers including physicians, pharmacists, advanced practice providers, and trainees. Participants were recruited via convenience sampling using X and professional listservs during May and June 2023. We solicited steward and program demographics and responses to statements exploring bias and discrimination through a 67-item electronic survey (Qualtrics). We further explored these experiences through semi-structured interviews.

**Results:**

Of 211 responses, 204 participants were included. Approximately half had been practicing for 5 years or less, 65% identified as female, and 24% identified as nonwhite or multiracial. Half of female stewards (50%) reported experiencing bias or discrimination in their role as an antimicrobial steward compared to 26% of male stewards. When controlling for race and ethnicity, seniority, and credentials, females were 2.8 times more likely (95% CI, 1.5–5.4; *P* < 0.01) to have experienced bias or discrimination when performing stewardship duties. Themes from our 16 interviews illuminated sources of perceived bias against stewards, the impact they had, and strategies to mitigate the influence of these biases.

**Conclusions:**

Bias and discrimination are felt disproportionately by women and junior antimicrobial stewards and can lead to poor job satisfaction and a lack of perceived effectiveness. Acknowledging these experiences and equipping stewards with strategies to mitigate their effects should be a priority of institutions and professional societies.

## Introduction

Bias against women and those underrepresented in medicine exists across the healthcare landscape,^1,2^ including in the practice of infectious diseases.^3^ Antimicrobial stewardship, the practice of promoting optimal use of antimicrobial agents to combat antimicrobial resistance, may be a venue for these biases to manifest. In the conduct of antimicrobial stewardship, gender and professional hierarchy can be a barrier to effective communication.^4–8^ For example, women stewards are less likely to have their recommendations accepted,^5^ which may risk suboptimal patient and health system outcomes and could contribute to negative feelings among these stewards.

While evidence supports differential identity-based experiences among stewards, the impacts on our stewardship workforce have not yet been described. Highlighting these experiences and urging interventions to mitigate bias and discrimination in the practice of stewardship may help curtail possible burnout among stewards.^9–11^

In this study, we sought perceptions of bias and discrimination among antimicrobial stewardship providers and, together with our participants, offer strategies to support our workforce in mitigating the impact of these experiences.

## Methods

### Participants and setting

A wide range of currently practicing antimicrobial stewards in the United States, including physicians, pharmacists, advanced practice providers, and trainees, were included.

### Survey design and distribution

J.T. and L.T. developed the survey, which was refined iteratively through expert consultation with the University of Wisconsin Survey Center and beta testing by 3 stewards, and administered electronically (Qualtrics, Provo, UT). A complete copy is provided in the supplemental materials. We solicited demographics, descriptions of antimicrobial stewardship programs (ASPs), and responses to statements regarding the experience of bias and discrimination in the conduct of stewardship using Likert scale responses. Participants were recruited through convenience and snowball sampling strategies: via an anonymous link by J.T. and L.T. personal X (formerly Twitter) accounts amplified by #IDTwitter community members, direct email to our ASP, encouragement of our colleagues to distribute widely via email, and through the American College of Clinical Pharmacy organization’s listserv. The survey was open from May 25 to June 30, 2023.

### Statistical analysis

Descriptive statistics summarized responses to demographic items. Gender was categorized into a binary variable as no respondents identified as non-binary. Race and ethnicity were categorized as a binary variable (ie, white and nonwhite) given the small proportion of nonwhite respondents. Years in current level of training were categorized into a binary variable of junior and senior (0–5, 6+ years), corresponding to the typical promotion timeframe. To explore the reported frequency of bias and discrimination, we developed a generalized linear model with a logit link function (ie, logistic regression) and a series of analysis of variance (ANOVA) models with a response variable of survey responses (5-point Likert scale) and explanatory variables of gender, race and ethnicity, and years in current level of training. To quantify the sources of bias, we developed a series of ANOVAs with a response variable of survey responses (7-point Likert scale) and constructed independent models with sole explanatory variables of gender, race and ethnicity, or years in current level of training. We used a Bonferroni correction to account for multiple comparisons (N = 9) when testing for steward characteristics perceived influence on interpersonal interactions. Significance for all models was assessed with an alpha of 0.05.

### Semi-structured interviews

We developed a semi-structured interview guide through an iterative process informed by consultation with the University of Wisconsin-Madison Institute for Clinical and Translational Research and 2 pilot interviews. The final guide is available in the supplemental material. We conducted semi-structured interviews among a volunteer subset of survey respondents who reported bias and discrimination. Interviews continued until data saturation, when only redundant data were being collected. Interviews were conducted by L.T., J.T., and A.G. via a secure web-based platform and recorded for transcription.

### Qualitative analysis

**Figure 1.**
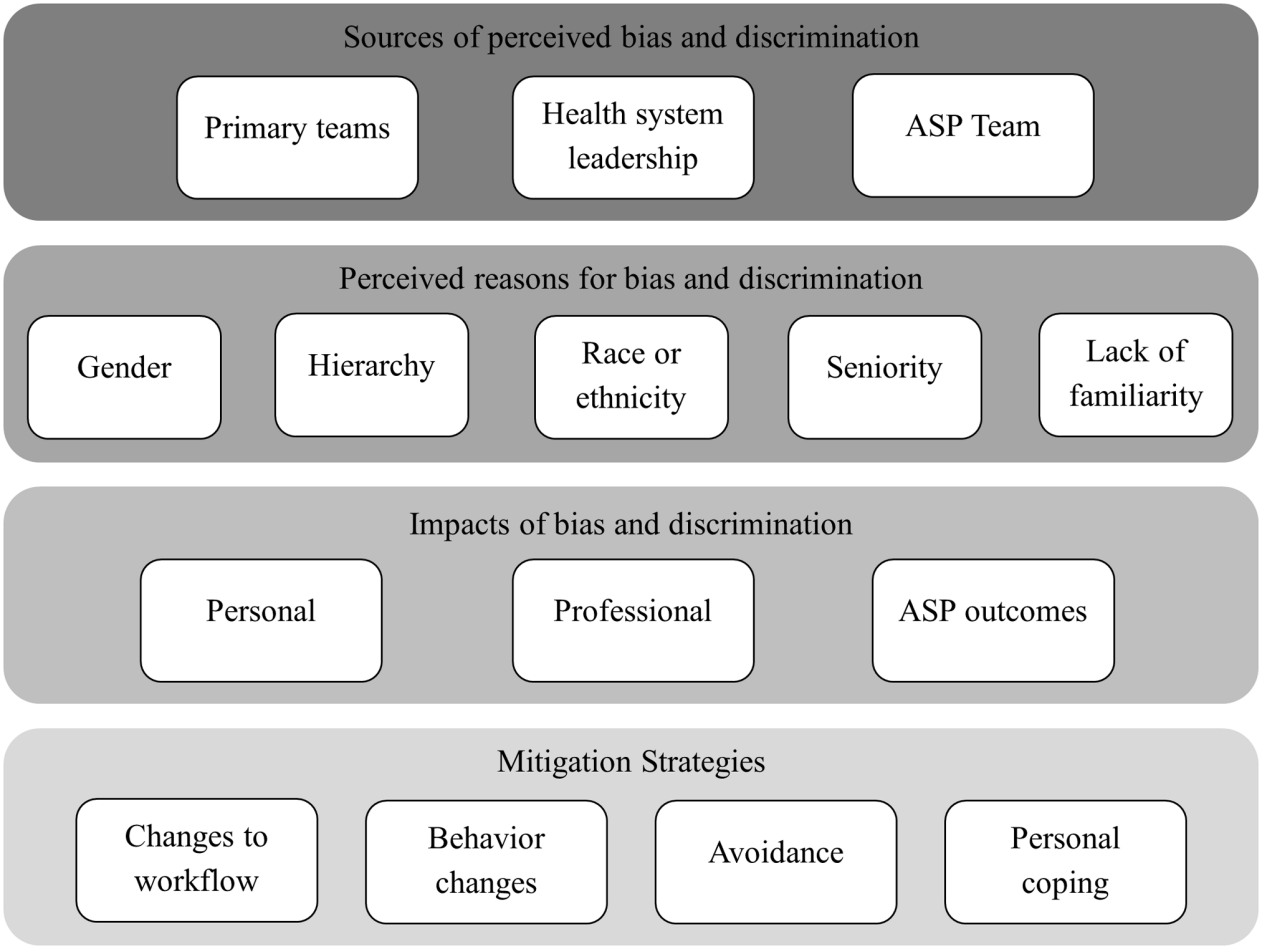
Sources of perceived bias, impacts on stewardship providers and programs, and mitigation strategies employed to lessen these impacts.

We applied an a priori coding scheme (Figure 1) informed by the themes in our interview guide and refined iteratively by 2 study team members during consensus coding (J.T. and L.T.). We offered the opportunity for member checks with interviewees. All qualitative data were managed using NVIVO software (QSR International Pty Ltd., Version 14, 2023). The included quotes were lightly edited for readability without changing the intent of the quote.

### Ethics

Our work was approved by the University of Wisconsin – Madison Health Sciences Institutional Review Board. Five randomly selected survey participants received incentives; all interviewees were compensated for their time.

## Results

### Survey respondent and interviewee characteristics

We received 211 survey results; 7 survey records were excluded for nonsensical responses. The median age of respondents was 37 years (IQR 32–43 years), 65% (n = 132) identified as female, and 24% (n = 49) identified as nonwhite or multiracial. Respondents had been practicing for a median of 7.5 years (IQR 3.8–12.0) at their current level of training. Practice settings spanned urban to rural and both teaching and non-teaching (Table 1). Most stewards reported their stewardship recommendations were sometimes challenged (55%, n = 123) but ultimately enacted most of the time (76%, n = 165). Nearly all (96%) communicate directly with primary teams using a variety of modalities (Table 1). All responses are provided in Supplemental Table 1.

**Table 1.** Stewardship provider and program characteristics

Steward characteristics	All n (%)	Female n (%)	Male n (%)
All respondents	204 (100)^a^	132 (65)	72 (35)
Age			
< 35	80 (39)	53 (40)	27 (38)
35 to <45	81 (40)	51 (39)	30 (42)
45 to <55	27 (13)	17 (13)	10 (14)
≥ 55	13 (6)	8 (6)	5 (7)
Race			
Asian	30 (15)	20 (15)	10 (14)
Black or African American	3 (1)	3 (2)	0 (0)
Hispanic/Latino	4 (2)	2 (2)	2 (3)
Middle Eastern or North African	1 (1)	1 (1)	0 (0)
White	155 (76)	101 (77)	54 (75)
Multi-race	11 (5)	5 (4)	6 (8)
Profession			
Pharmacist	154 (75)	98 (74)	56 (78)
Physician	34 (17)	21 (16)	13 (18)
Nurse	6 (3)	5 (4)	1 (1)
Advanced practice provider	2 (1)	2 (2)	0 (0)
Pharmacy resident	6 (3)	5 (4)	1 (1)
ID fellow	1 (1)	1 (1)	0 (0)
Full-time equivalent (mean (SD))	0.53 (0.41)	0.51 (0.45)	0.57 (0.32)
Number of stewards in team (mean (SD))	4.7 (3.5)	5.1 (3.8)	4.0 (2.6)
Years practicing at current level of training			
≤ 5	105 (51)	65 (49)	27 (37)
6–10	63 (31)	45 (34)	23 (32)
> 10	36 (18)	22 (17)	22 (31)
Location of practice			
Northeast	48 (24)	35 (27)	13 (18)
Southeast	44 (22)	30 (23)	14 (19)
Midwest	63 (31)	38 (29)	25 (35)
Southwest	24 (12)	16 (12)	8 (11)
West	17 (8)	9 (7)	8 (11)
Northwest	8 (4)	4 (3)	4 (6)
Program Characteristics			
Setting^b^			
Urban, teaching acute hospital	123 (60)	76 (58)	47 (65)
Urban, non-teaching acute hospital	40 (20)	25 (19)	15 (21)
Suburban, teaching acute hospital	48 (24)	32 (24)	16 (22)
Suburban, non-teaching acute hospital	41 (20)	27 (20)	14 (19)
Rural, teaching acute hospital	27 (13)	18 (14)	9 (13)
Rural, non-teaching acute hospital	44 (22)	29 (22)	15 (21)
Long-term acute care hospital	21 (10)	12 (9)	9 (13)
Long-term care facility	21 (10)	12 (9)	9 (13)
Ambulatory clinics	50 (25)	36 (27)	14 (19)
Patient population			
Adult (≥19 years)	132 (65)	80 (61)	52 (72)
Pediatric (≤18 years old)	21 (10)	15 (11)	6 (8)
Both	51 (25)	37 (28)	14 (19)
Age of stewardship program			
≤ 5	40 (20)	29 (22)	11 (15)
6–10	90 (44)	55 (42)	35 (49)
> 10	73 (36)	47 (36)	26 (36)
Methods of conducting stewardship^b^			
Preauthorization	141 (69)	93 (70)	48 (67)
Prospective audit and feedback	183 (90)	117 (89)	66 (92)
Face-to-face stewardship	112 (55)	72 (55)	40 (56)
Electronic medical record-based interventions	186 (91)	119 (90)	67 (93)
Facility-specific treatment guidelines	184 (90)	116 (88)	68 (94)

a Incomplete responses included may not sum to 204 in each category.

b All settings and modalities in which stewardship is provided were indicated, which may be more than one per respondent.

Sixteen semi-structured interviews were conducted with pharmacists (n = 13) and physicians (n = 3), 9 of whom identified as women and 7 identified as men, 11 identified as white, 2 as Asian, and 2 as Middle Eastern or North African.

### Experience of perceived bias

**Figure 2.**
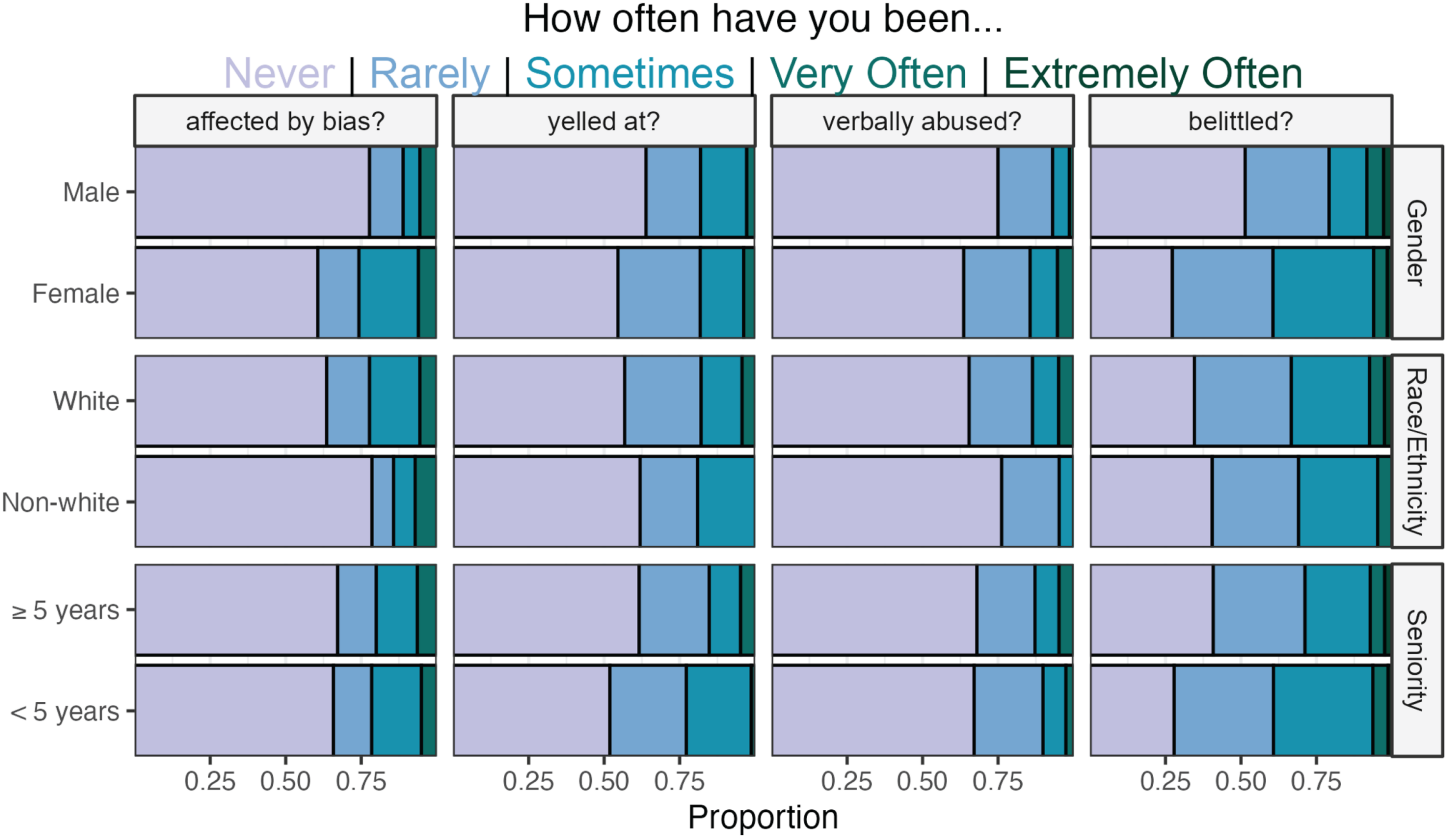
Frequency of stewardship providers’ experience of bias, stratified by gender, race/ethnicity, and seniority. Colors denote a 5-point Likert response.

Nearly half of the respondents (42%) reported experiencing bias or discrimination in their role as antimicrobial stewards. Females (50%) were nearly twice more likely than males (26%) to report experiencing bias (Figure 2). When controlling for race and ethnicity, seniority, and credentials, female stewards were 2.8 times more likely (95% CI, 1.5–5.4; *P* < 0.01) to report bias or discrimination during stewardship duties. Females reported they were more frequently verbally abused (mean difference = 0.23, *P*-value < 0.05) or belittled (mean difference =0.4, *P*-value < 0.01) when controlling for race/ethnicity and seniority. The frequency of these experiences did not differ by race/ethnicity or seniority.

Interviews illuminated sources of perceived bias against stewardship clinicians, the impact they had on individuals and programs, and strategies employed to mitigate the influence of these biases (Figure 1). Exemplary quotes are included for additional context (Tables 2 and 3).

**Table 2.** Exemplar quotes from semi-structured interviews of sources and impact of bias

Sources of perceived bias	
Gender	“[They] didn’t appreciate recommendations necessarily from a female. But if one of my other clinical specialist colleagues […] would […] suggest the exact same thing, it might be accepted from him but not from necessarily me.” – Female, pharmacist “he came barging in, ‘who are you to tell me I can’t discharge my patient?’ […]And on one end, I feel like it probably came down to like, oh, a pharmacist is telling me that a physician can’t discharge a patient[…] But then also, I know his past interactions, so it’s also like a woman is coming to him and is telling him you can’t do X, Y, Z […]” – Female pharmacist describing an attempt to intervene with previously unrecognized Staphylococcus aureus bacteremia
Professional hierarchy	“I got told, ‘I’m the physician, not you’. This was on the phone, and they hung up on me.” – Female, pharmacist, when providing antibiotic recommendations
Seniority	“I guess when I was a fellow doing it, I imagine the fellows would probably agree, like they’re probably a little less willing to contact the attending. And even as a junior stewardship member, I feel less confident contacting the attending.” – Male, physician “I feel like the bar is relatively low to be critical of something that I say at a stewardship meeting as I compare that to other people who are certainly more senior.” – Male, physician
Race and ethnicity	“One of my very good colleagues is a Muslim female who has shared with me that she has felt that she has been discriminated not only against her gender but perhaps her culture or religion, if you will, that she has been treated differently compared to how she’s seen a provider interact with other pharmacists.” – Female pharmacist reflecting on a colleague’s experience
Lack of familiarity	“I was the newcomer. I didn’t necessarily feel like the recommendations were being taken as seriously or being acted on as much as I perceived they were from what I had seen with [predecessor].” – Female, pharmacist “I think, as many things are, stewardship is relational […}if I don’t know them. […] they don’t know my name when I send it out to them, or at least I don’t perceive that they do, and I don’t know how well my recommendations are trusted as a result of that lack of sort of relational component.” – Male, physician

Impact of bias	
Personal	“Being asked to do more and the way that others treat you when you’re not even feeling effective at your own job because of some of these interactions is a big dissatisfier and I think contributes overall to your well-being in a negative way and explains probably why so many people decided to leave ID and go to industry.” – Female, pharmacist “It’s frustrating. It’s, I mean, frankly, it feels disrespectful. You know, we’ve worked hard for this and feel pretty confident in our ability to manage antibiotics and treat infections. And to sort of have that disregarded or seen as unimportant or, it just, like you wish that people would be a little bit more willing to consider our expertise.” – Male, physician
Professional	“I feel I should have the opportunity to make an impact because there’s residents here and a lot of opportunities for teaching and exploring, carrying on the future of stewardship. But it just felt like I was being limited.” Female, pharmacist “[…] in my one-on-ones and evaluations with him, or when I would go to him with a concern, he actually told me once that he felt like he was managing kindergarteners. And so just that lack of kind of support and the feeling of, well, I can’t do anything to prove it really affected my job satisfaction and mental health, and that was ultimately what led to my decision to leave.” – Female pharmacist reflecting on challenging dynamics with a supervisor
Patient safety and program outcomes	“[…] after several rejections from the same team on the same sort of issues, I think they sort of know that we’re going to call, and then they just don’t. They know they’re going to come up with an answer to not make a change […] which, of course, is unfortunate because those are usually the areas of greatest need for intervention.” – Male, physician “I think any delays in going to what is the best or implementing the best option in terms of a policy, a protocol, a guidance document, etc., any delays there are going to have negative impacts on patients and patient care.” – Male, pharmacist

**Table 3.** Exemplar quotes on bias mitigations strategies and sources of support

Strategies to mitigate bias	
Changes in workflow	“One thing I recognize as super effective, I’m just not able to implement that personally myself here, is […] in-person rounding on different units. […] almost like handshake rounds where it’s like, ‘hi, like look me in the eye and reject me now’.” – Female, pharmacist “Beef up the recommendation next time or cite an article. […] now a lot of my note templates have […] references in them with links.” – Female, pharmacist “EMR-based strategies, I would say, are probably the strongest one. If you’re forcing people to just do things, then that’s probably the ultimate least confrontational way where you’re going to avoid some of these things.” – Female, physician “I’ll start interventions that I know are like slam dunks. They should be […] really easy. Then I move on to the more challenging interventions as the day progresses just because why start out the day on a negative when I can start out the day more positively?” – Male, pharmacist
Avoidance of challenging situations	“I would say overall we spend less time in the ICU. Actually, particularly less in the medical ICU, interestingly, because […] it’s very time consuming for us, and we have very little impact.” – Female, physician “[…]law of diminishing returns. You can only put so much time and effort into something before it’s just making it worse, and you’re taking away from time for other things.” – Female, pharmacist
Behavior changes	“[…]she was able to talk their language in the sense, not necessarily technical language but completely informal. She sounded like one of the boys for a second. She was able to code switch entirely to fit in.” - Female pharmacist commenting on observed behavior of a more senior, female colleague + “[…] I always try to word it in a collaborative and respectful manner that also still leaves the autonomy in the providers’ place… And then there’s a couple questions that you can ask at the end. Like, do you see this differently? Or, am I missing something here, or is there something I don’t understand?” - Male, pharmacist
Personal coping strategies	“[…] I had to do like kind of a mental pep talk and psych up that I’m, okay, I need to make these recommendations for the patient. It’s in their best interest to really get myself motivated to initiate that interaction.” – Female, pharmacist

Sources of support	
Interpersonal	“[…] we now have a male ID physician. And I will say, since we’ve had him come in, it’s like he fits into the boys’ club so nicely. He is good in that he sees that, and he paves ways for his colleagues, which we appreciate.” – Female, pharmacist “[…] my primary mentor is the medical director of the stewardship program…. We meet every week. […] It’s not uncommon that people bring up challenging things. And I think that provides […] an opportunity to share frustrations and know that we’re not alone.” – Male, physician
Institutional	“Communicating who we are, what our services are, I think would have helped, especially in the beginning. Or as even now as we’re getting, you know, new providers just kind of almost advertising the stewardship team more, maybe providing more resources on what we offer.” – Female, pharmacist “I wonder if just more leadership support from the departments and services that we’re interacting with frequently would be helpful. […] if the Department Chair of Hospital Medicine was more vocally, or more outspoken in support of stewardship in general […] maybe their colleagues in that department would be more receptive to stewardship interventions?” – Female, pharmacist
Professional organizations	“[…] if other people had useful things that work for them, like having that collated somewhere as a toolkit, could be useful.” – Male, pharmacist “[…] funding research like your research to just to learn more about this topic and maybe more of the psychologic or implementation science side of things. Cognitive biases and that sort of thing, of how we, how do we actually not only recognize that these biases exist but do something about them and to change these outcomes. And, is there some sort of behavioral science curriculum that should be incorporated into ID training or stewardship training?” – Female, pharmacist

### Sources of perceived bias

**Figure 3.**
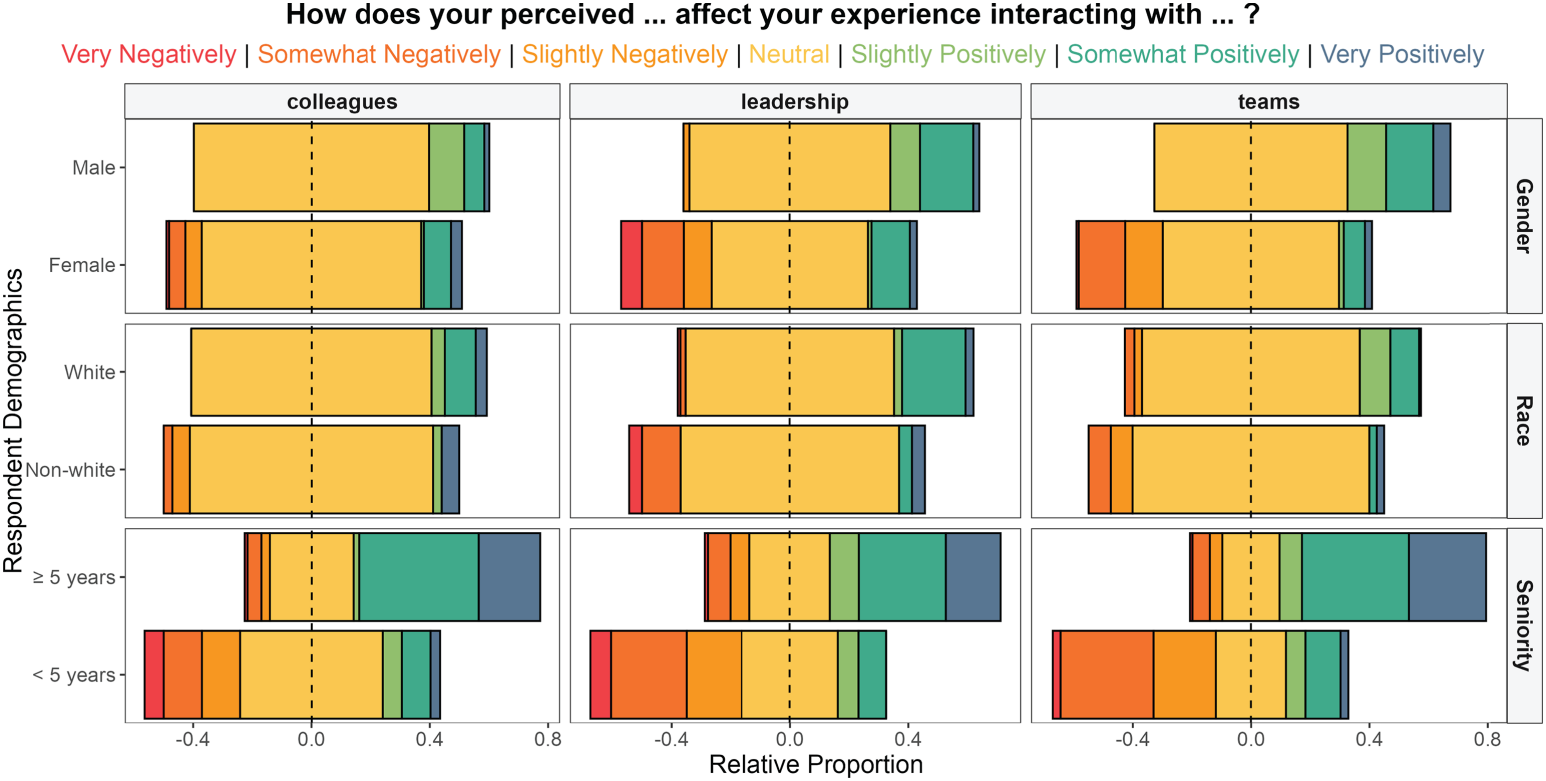
Steward perceptions of gender, race, and seniority on interactions as a stewardship provider with primary teams, stewardship colleagues, and healthcare leadership. Colors denote a 7-point Likert response, and proportions are centered on a neutral response.

Female stewards more frequently reported that their gender negatively affects their interactions with primary teams (F_1,193_ = 28.4; *P* < 0.001), stewardship colleagues (F_1,166_ = 1.7; *P* < 0.05), and leadership (F_1,133_ = 11.3; *P* < 0.001) compared to male stewards. Nonwhite stewards more frequently reported that their race and ethnicity negatively affect their interactions with primary teams (F_1,193_ = 5.06; *P* < 0.01), stewardship colleagues (F_1,166_ = 3.0; *P* < 0.01), and leadership (F_1,133_ = 7.0; *P* < 0.05) compared to those identifying as white. Junior stewards reported that their level of seniority negatively influences their interactions with primary teams (F_1,193_ = 76.6; *P* < 0.001), stewardship colleagues (F_1,166_ = 44.2; *P* < 0.001), and healthcare leadership (F_1,166_ = 34.6; *P* < 0.001) compared to senior stewards (Figure 3).

Nearly all interview participants perceived bias in discussions with primary teams, several in their interactions with health system leaders, and a few with members of their stewardship team (Table 2). Age and perceived junior status are believed to play the largest role in these interactions; professional hierarchy and gender were also commonly cited as perceived sources of bias. Race and ethnicity were mentioned infrequently.

Most stewards described instances when recommendations were dismissed or met with hostility by primary teams, who cited more experience or authority. The provided rationale was often more years of experience or perceived greater authority of physicians over pharmacists, despite several stewardship pharmacists feeling armed with more contemporary information to guide antimicrobial choices. Some stewards reported being screamed at or humiliated. Female stewards noticed their recommendations were more accepted when presented by male colleagues. Positive interactions were more common with familiar clinicians, trainees, and pharmacists.

When interacting with health systems representatives, such as during pharmacy and therapeutics or guidelines committee meetings, several stewards perceive their opinion to be disregarded owing to their perceived age. Pharmacists perceive their opinion to be less influential than physicians when working at the hospital or system level. Stewards in their positions during the emergence and height of the COVID-19 pandemic felt their expertise came to be valued due to the sheer volume of work they did to develop guidelines and allocate resources.

Regarding experiences of bias and discrimination when interacting with ASP colleagues, women cited the need to “prove themselves” before their opinions were valued. Junior stewards, regardless of gender or profession, felt their opinions were less valued than senior colleagues.

### Impact of bias

**Figure 4.**
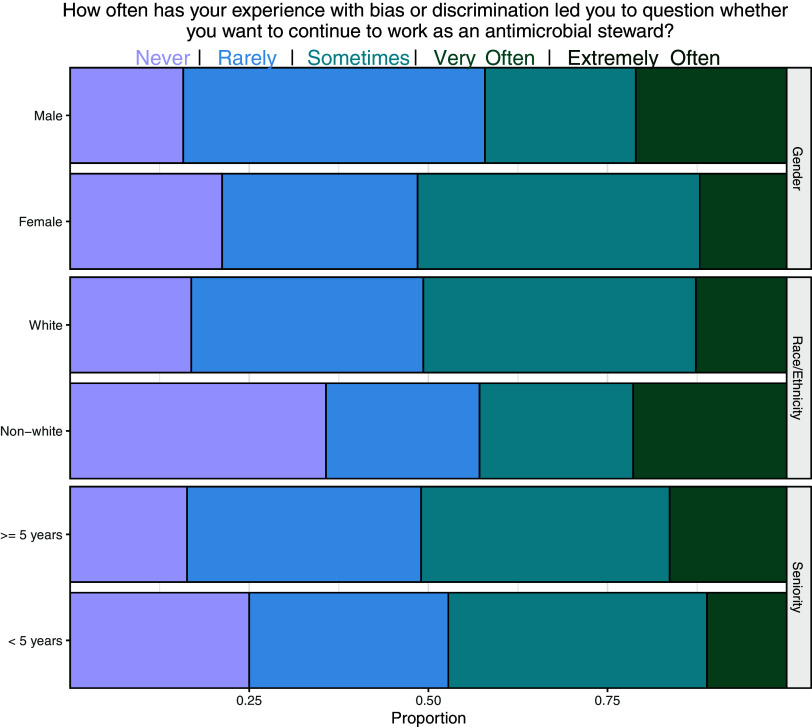
Frequency of thoughts of leaving stewardship in stewards who perceive bias and discrimination in their role stratified by gender, race/ethnicity, and seniority. Colors denote a 5-point Likert response.

Stewards who perceived bias reported lower satisfaction in their stewardship duties (F_1,202_ = 6.59; *P* = 0.01) and more frequent recurrent negative thoughts about interactions with primary teams (F_1,202_ = 18.76; *P* < 0.001) than those who did not perceive bias. Four of 5 respondents who experienced bias (80%, n = 68/85) shared that these experiences led them to question if they wanted to continue stewardship work, with similar responses across demographics (Figure 4). Several interviewed stewards echoed these sentiments, with 2 leaving previous positions.

Many stewards have difficulty processing these experiences of bias and discrimination and an emotional impact that extends into their non-work life. The cumulative stressors with primary teams and the health system contribute to a lack of perceived effectiveness and worsening of well-being for some stewards. Many struggled with their expertise being dismissed when they see suboptimal antimicrobial decision making that could negatively impact patient care. Several stewards reported that processing difficult interactions can take days and are associated with mental exhaustion that can impair their non-work life. This can lead to questioning of their own knowledge and value.

Several stewards expressed concerns about how biases against them can lead to poor patient outcomes. Delays in optimal antimicrobial prescribing, difficulty advancing institutional policies, protocols and guidance, and delays in acquiring additional resources for the ASP are perceived to negatively influence the effectiveness of the ASP.

### Strategies to mitigate bias and discrimination

Stewards described several different strategies to mitigate the negative impacts they perceived (Table 3). Most stewards adjust their workflow for challenging interactions. Junior and women stewards often include senior colleagues in communications to mitigate anticipated pushback on recommendations and guard against unprofessional responses. In situations where they anticipate resistance, some women and junior stewards funnel recommendations through male or senior colleagues or through team pharmacists who have better rapport with prescribers.

Many stewards heavily rely on citing guidelines or relevant primary literature to justify their recommendations. A tendency to intensely prepare for or rehearse discussions was often described. Some stewards overcame a pattern of difficulty communicating with primary teams by increasing their face-to-face time with them, while others find electronic communication provided less opportunity for bias and discrimination to influence discussions. Built-in electronic medical record-based interventions are perceived by some stewards to maximize stewardship interventions while minimizing the potential for difficult interactions.

Many stewards delay or avoid challenging interventions altogether. Most survey respondents rarely (32%, n = 65) or sometimes (44%, n = 90) avoided challenging conversations with primary teams. Some described starting their day with “easy wins.” Commonly, stewards avoid interactions with teams or settings in which they anticipate being less effective to efficiently allocate their effort.

Some stewards, particularly those junior and women, describe changes to their behavior to avoid the impact of bias in their encounters. Some use a less assertive approach to conversations they expect to be difficult by asking questions to guide the prescriber instead of providing a recommendation. In interactions with larger groups or health systems representatives, many stewards explicitly invoke their credentials and change their vocal tone, posture, and hand gestures to avoid appearing young or feminine.

To cope with distressing interactions and job-related stress, many stewards debrief with their colleagues, meet regularly with mentors, and seek counseling to process these experiences. Several incorporate exercise and meditation to process work-related stress.

### Sources of support

Mentorship is an important source of support for stewards, with 40.2% (n = 82) reporting access to ongoing mentorship, which is perceived as adequate by 87.8% (n = 72). In addition to advice from mentors, most stewards sought support from their colleagues. Some were fortunate to have individual allies in hospital leadership to whom they could escalate issues. Institutional endorsement and promotion of the work of ASPs validate stewards and give them greater confidence. Networking and contact with colleagues from other institutions facilitated by national societies is helpful to some. Many stewards feel professional meetings can be leveraged to have open forums or training sessions on how to approach challenging discussions and mitigate bias and discrimination in our work.

## Discussion

Stewards perceived bias and discrimination based on several aspects of their identity, most prominent in our study being junior, female, nonwhite, and at a disadvantage in the traditional physician–pharmacist professional hierarchy. A troubling number of women stewards report verbal harassment and belittlement when interacting with prescribers. These negative experiences may contribute to a decreased sense of effectiveness and pride and lower job satisfaction reported among early-career and women stewards and can have personal, professional, and programmatic consequences that risk harm to patients. These findings should trouble those concerned about the future of our stewardship workforce, which has been under increasing strain since the onset of the COVID-19 pandemic.^9–12^

While not unique to our field, stewardship is not protected from bias against women and gender minorities, complicated further by perceived professional hierarchy. In a study conducted by Vaughn *et al*, women pharmacists without specialty training in infectious diseases were less likely to have their antibiotic stewardship recommendations accepted during antibiotic time out close to the time of discharge compared to male pharmacists.^5^ In contrast, a larger study conducted by Ausman *et al* did not demonstrate a statistically significant difference in the acceptance of stewardship interventions via prospective audit and feedback between female and male stewardship clinicians.^13^ These discordant findings may in part be related to differences in the level of infectious disease specialty training, timing and type of stewardship interventions, and site or program-specific factors. Although our study design did not allow for assessing stewardship practice outcomes, interviewed women stewards in our study perceived their expertise to be challenged more often and their opinion to be less influential, consistent with empirical evidence of the role of women experts in group settings.^14,15^

The perceived professional hierarchy between physicians and pharmacists that contributes to negative interactions with primary teams and health systems is well described.^8,16,17^ The tension between physicians as prescribers and pharmacists as drug experts is often felt in antimicrobial stewardship, given the frequency of pharmacists providing advice to physicians.^6–8,16,17^ The sources of greatest perceived difficulty among our interviewees were senior, male providers, which is consistent with prior work examining uptake of stewardship recommendations.^18,19^ As in our study, this tension leads to avoidant behaviors among stewardship pharmacists.^4,8,17^ Reliance upon external validation for recommendations or through practice standards or other guidance is also described as a way to avoid tension in a discussion with prescribers.^6,8^

Although stewards identifying as a racial or ethnic minority reported experiencing bias at a similar rate and intensity as white stewards, they reported that they perceived their racial and ethnic identities to negatively impact their interactions with primary teams, their stewardship colleagues, and hospital leadership. This is consistent with reported perceptions of bias of minority physicians.^20^ Additionally, it is possible that nonwhite stewards experienced microaggressions that were not captured in the survey because they were not considered to represent “abuse” or “belittlement.”^21^ It is also possible that electronic and phone communication masks racial and ethnic identities in a way that it cannot mask gender identity. Further, by isolating race and ethnicity in our analysis, we risk oversimplifying the influence of intersectionality that likely impacts the experience of all stewards.

Stewards in our interview sample employ strategic changes in their communication styles to avoid provoking defensive responses and face-to-face interactions to facilitate partnerships with prescribers, which are behaviors described in other work to promote collaboration between stewards and prescribers.^4,22^ As in prior work, stewards in our study who perceived an “us versus them” mentality often deferred to hierarchy and avoided face-to-face interactions and had lower satisfaction with their work in stewardship.^4^ Although our sample skewed toward junior stewards, it is likely that communication strategies evolve with experience. Expanding mentorship and specific communication training early in a career may help accelerate the acquisition of these skills.

There are limitations to this study. We are unable to calculate a response rate due to the use of convenience sampling, limiting the generalizability of our findings. The use of X/Twitter to recruit respondents in 2023 may have selected a progressive subset of the stewardship community. Our survey distribution method may have skewed responses toward overrepresenting the perspectives of pharmacists relative to other antimicrobial stewardship clinicians, and recruitment language likely attracted those with relevant experiences. Interview recruitment was reliant upon those responding to the survey, so this overrepresentation may have persisted in our interview sample. However, pharmacists account for much of the stewardship workforce and are an important voice to amplify as we aim to achieve equity and inclusion in our work. Our sample included a small proportion of stewards of racial or ethnic minority groups; dichotomizing race oversimplifies the heterogeneity of experiences by individuals of different or overlapping minoritized racial and ethnic identities. Interviews were conducted by white women physicians, and we acknowledge this may have influenced the psychological safety of some interviews and, by extension, our results. Further, we acknowledge our positionality may have biased our interpretation of qualitative results; however, we offered member checks to mitigate the biases we may bring to the analysis.

We must combat the threats to well-being, satisfaction, and effectiveness of our stewardship workforce. Health system leadership can support our stewards by promoting credibility through endorsing the important work of ASPs. Our professional organizations can serve as key forums for stewards to provide interpersonal support and strategize on how to overcome barriers to satisfaction and effectiveness. We also call on our professional organizations to develop resources for stewards desiring additional professional development in negotiating difficult interactions and those looking to advance equity and inclusion in their institutions and professions.

## Supporting information

Tischendorf et al. supplementary material 1 Tischendorf et al. supplementary material

Tischendorf et al. supplementary material 2 Tischendorf et al. supplementary material

Tischendorf et al. supplementary material 3 Tischendorf et al. supplementary material

## Supplementary material

The supplementary material for this article can be found at https://doi.org/10.1017/ice.2025.10224.
