# Supplementary material for: Bias and discrimination perceived by antimicrobial stewards: a mixed-methods study
Source: Infect Control Hosp Epidemiol. 2025 Aug 19;46(9):910–9. doi: 10.1017/ice.2025.10224 (PMC12616225; doi:10.1017/ice.2025.10224)
Supplement: Tischendorf et al. supplementary material 1 — Tischendorf et al. supplementary material [file S0899823X25102249sup001.docx]

**Semi-structured interview Guide**

Participant info (filled in by study team based on screening survey):

Name

Profession

Gender identity

Racial/ethnic identity

Age

**Introduction**

My name is [XXX] and I am an assistant professor in the Division of Infectious Disease at the University of Wisconsin-Madison. I’m part of the antimicrobial stewardship program. Our goal for this study is to explore experiences and impact of bias and discrimination among stewardship providers and to discuss strategies you use to mitigate the effects of this bias in your work. We will record this interview and any identifiers (specifically your name, institution name) will be removed from the transcript. With your consent we would like to proceed with the interview and will start the recording.

(**PRESS RECORD BUTTON NOW**)

1. Tell me why you wanted to participate in this interview.

**Understanding logistics**

1. Tell me about a typical week in service of stewardship.
2. What activities are you completing?
3. Who are you speaking to?

**Bias and discrimination**
Research has demonstrated the presence of gender, racial and ethnic based bias and discrimination across medicine, including in the practice of antimicrobial stewardship. You indicated in your screening survey that you have experienced bias and discrimination in your role as an antimicrobial steward.

1. Tell me about your experience of bias or discrimination in execution of your duties as a stewardship provider. Please provide specific examples. (ask probes about each incident separately)
   1. Who are they with? (primary teams, colleagues, health system personnel?)
   2. When do they occur? (interactions with primary teams, during AS meetings?)
   3. How did this experience make you feel?
2. How does gender play a role in your experience?
3. How does race/ethnicity play a role in your experience?
4. How does seniority play a role in your experience?

*Probe: what other memorable experiences would you like to share?*

Some of us experience bias and discrimination as an annoyance, but others find it a major impediment to our well-being, satisfaction, and effectiveness.

1. Describe the effect these experiences have on you.
   1. On your effectiveness as a stewardship provider
   2. On your professional satisfaction.
   3. On your well-being (probe: others have described the extra mental load)

**Mitigation**

Many individuals who experience bias and discrimination in the professional setting have developed strategies to mitigate the influence on their work and well-being. We’re interested in understanding how you manage these experiences.

1. Describe the strategies have you employed to mitigate the influence of bias or discrimination in your work an antimicrobial stewardship provider.
   1. How might you change your behavior depending on the interaction you are having?
   2. What strategies do you use when communicating with primary teams?
   3. What strategies do you use when communicating with your colleagues?
   4. What strategies do you use when communicating with health system representatives?
   5. What strategies do you employ to take care of yourself? (especially if they have mentioned effects on their well-being)
2. Describe the strategies you’ve observed colleagues use.
   1. Do they change their behavior depending on the interaction they are having?
   2. What strategies do you observe when they are communicating with primary teams
   3. What strategies do you observe when they are communicating with your colleagues
   4. What strategies do you observe when they are communicating with health system representatives?

While one cannot know the true experience of another, many of us have witnessed bias and discrimination directed towards our colleagues (or learners).

1. Describe the bias or discrimination you’ve witnessed toward your colleagues.
   1. Who was involved in the incident?
   2. What is your colleague’s position, gender, race/ethnicity, seniority?
   3. Have you had conversations with your colleagues about their experiences? Please describe.
   4. How has this interaction affected your work environment?

You told me earlier [effect A, effect B, etc].

1. How could you best be supported in order to mitigate these effects and other adverse impacts of bias and discrimination?
   1. By your colleagues?
   2. By your institution?
   3. By national organizations?

**Closing**
What didn’t I ask about that is important for me to know about your experience?

Thank you for giving us your time and sharing your experiences today. We understand that talking about this can be difficult. I am sorry that these experiences occurred, and also empathize with them.
