# Supplementary material for: Bias and discrimination perceived by antimicrobial stewards: a mixed-methods study
Source: Infect Control Hosp Epidemiol. 2025 Aug 19;46(9):910–9. doi: 10.1017/ice.2025.10224 (PMC12616225; doi:10.1017/ice.2025.10224)
Supplement: Tischendorf et al. supplementary material 3 — Tischendorf et al. supplementary material [file S0899823X25102249sup003.zip › Sup/Appendix A Survey Tool.pdf]

Block 1

Thank you for your interest in our study.

We are interested in learning more about the burden of bias in our everyday antimicrobial stewardship duties. As a first step, we invite you to complete this online questionnaire, which should take about 20 minutes to complete. All of your responses to this questionnaire are confidential. Any information you share will only be reported in group form and will not be reported in a way that would allow you to be personally identified.

If you have any questions about participating, please contact Jessica Tischendorf (jtischen@medicine.wisc.edu) or Lindsay Taylor (ltaylor@medicine.wisc.edu, 608-263-1545).

Instructions to complete the questionnaire:

To choose a response, click on the button that corresponds to your answer. If you would like to change your answer, click on a different button. You may go back to earlier screens by clicking the 'BACK' button at the bottom of the screen. To advance to the next screen click the 'NEXT' button. Please try to complete this short survey in one sitting. If you start the survey and are unable to finish it, you can exit by simply closing the browser window. However, if you close your browser before finishing, when you return to the survey you will need to start again from the beginning because the survey will not retain your previous answers.

If there is a question you leave blank, you will be informed that it was left blank. You may then either return to answer that question, or continue on to the next question. You are not required to answer any question you would prefer to skip. After the entire questionnaire has been completed and all the data are ready to be submitted, please click on the 'SUBMIT' button on the last screen. Once you click 'SUBMIT' you will not be able to re-enter the questionnaire.

We appreciate your time and effort in assisting us by completing this web-based questionnaire. At the end of the survey, a link will bring you to a separate survey to share your contact information for gift delivery.

Please click 'NEXT' to begin the questionnaire now.

We recognize that many of you may be involved in more than one antimicrobial stewardship program. Are you currently working in two or more different antimicrobial stewardship programs?

- ○ Yes
- ○ No

Default Question Block

The first questions are about the characteristics of your antimicrobial stewardship program and your individual role. ${e://Field/Q1yes_Q2txt}

Does your antimicrobial stewardship program use each of the following modalities to conduct routine stewardship activities?

Does your program use…

	Yes	No
…preauthorization?	○	○
…prospective audit and feedback, sometimes called post-prescription review?	○	○
…face-to-face stewardship, such as handshake stewardship?	○	○
…electronic medical record-based interventions?	○	○
…facility specific treatment guidelines?	○	○
…education of treatment teams?	○	○
…other modalities? Please tell us: ____	○	○

In your program, are there other antimicrobial stewardship providers or do you practice independently?

- ○ There are other antimicrobial stewardship providers
- ○ I practice independently

How many antimicrobial stewardship providers share duties in your program?

[ ]

What is the profession of the institutionally recognized leader or leaders of your stewardship program?

Are they…

	Yes	No
…a pharmacist?	○	○
…a physician?	○	○
…a nurse?	○	○
…an advance practitioner provider, such as physician assistant or nurse practitioner?	○	○

	Yes	No
…other profession? Please tell us: ☐	○	○

Are you one of the institutionally recognized leaders of your stewardship program?

- ○ Yes
- ○ No

When was your antimicrobial stewardship program established?

- ○ Less than five years ago
- ○ Between five and ten years ago
- ○ More than ten years ago

Which of the following stewardship activities do you personally participate in?

Do you …

	Yes	No
…conduct prior authorization?	○	○

	Yes	No
…perform audit and feedback?	○	○
…participate in in-person interactions with primary teams, such as handshake stewardship rounds?	○	○
…provide program oversight?	○	○
…attend stewardship meetings?	○	○
…participate in stewardship-based quality improvement or scholarship?	○	○
…participate in the development of policies, guidelines, or protocols?	○	○
…participate in formal education of treatment teams, such as Grand Rounds or CME?	○	○
…participate in other activities? Please tell us: ______	○	○

What percentage of your time is dedicated to execution of your stewardship duties?

☐ ______ FTE

☐ Or, don't know

Do you currently conduct stewardship in each of the following settings?

	Yes	No
Urban, teaching acute care hospital	○	○
Urban, non-teaching acute care hospital	○	○
Suburban, teaching acute care hospital	○	○
Suburban, non-teaching acute care hospital	○	○
Rural, teaching acute care hospital	○	○
Rural, non-teaching acute care hospital	○	○
Long-term acute care hospital	○	○
Long-term care facility, such as skilled nursing facility	○	○
Ambulatory clinics	○	○
Other setting, please tell us: ______	○	○

Which one of the following is the primary setting at which you conduct stewardship?

- ○ Urban, teaching acute care hospital
- ○ Urban, non-teaching acute care hospital
- ○ Suburban, teaching acute care hospital
- ○ Suburban, non-teaching acute care hospital
- ○ Rural, teaching acute care hospital
- ○ Rural, non-teaching acute care hospital

- ◯ Long-term acute care hospital
- ◯ Long-term care facility, such as skilled nursing facility
- ◯ Ambulatory clinics
- ◯ ${q://QID12/ChoiceTextEntryValue/10}

For which patient population do you conduct stewardship activities?

- ◯ Adult patients, 19 years old and older
- ◯ Pediatric patients, 18 years old and younger
- ◯ Both adult and pediatric patients

The next questions are about your interaction with primary teams. When we ask about "primary teams" we are referring to teams who prescribe antimicrobials to patients for whom you are providing stewardship duties. ${e://Field/Q1yes_Q13txt}

As part of your current stewardship duties, do you communicate directly with primary teams either verbally, by text or electronically?

- ◯ Yes
- ◯ No

In the last year, how much of the time did you communicate with primary teams through each of the following modalities?

How much of the time did you communicate through…

	Never	Rarely	Sometimes	Most of the time	Always
…asynchronous notes in medical record?	○	○	○	○	○
…text messaging?	○	○	○	○	○
…electronic chat within electronic health record?	○	○	○	○	○
…telephone or video-based application?	○	○	○	○	○
…in-person?	○	○	○	○	○

In situations requiring direct communication with primary teams, either verbal, text or electronic, in the last year, how often were your antimicrobial stewardship recommendations challenged?

- ○ Never
- ○ Rarely
- ○ Sometimes
- ○ Most of the time
- ○ Always

Overall, when interacting with primary teams, in the last year, how often were your recommendations as an antimicrobial stewardship provider ultimately enacted?

- Never
- Rarely
- Sometimes
- Most of the time
- Always

Compared to other stewardship providers at your institution, in the last year how often were your recommendations challenged?

- A great deal less often
- Somewhat less often
- Slightly less often
- About as often
- Slightly more often
- Somewhat more often
- A great deal more often

While conducting your duties as an antimicrobial stewardship provider, how do you feel your perceived <u>gender identity</u> influences your experiences interacting with primary teams?

- Negatively

- Positively
- Not at all

How ${q://qid21/choicegroup/selectedchoices} do you feel your perceived gender identity influences your experiences interacting with primary teams?

- Slightly
- Somewhat
- Very

While conducting your duties as an antimicrobial stewardship provider, how do you feel your perceived racial or ethnic identity influences your experiences interacting with primary teams?

- Negatively
- Positively
- Not at all

How ${q://qid24/choicegroup/selectedchoices} does your perceived racial or ethnic identity influence your experiences interacting with primary teams?

- Slightly
- Somewhat
- Very

While conducting your duties as an antimicrobial stewardship provider, how do you feel your perceived level of seniority influences your experiences interacting with primary teams?

- Negatively
- Positively
- Not at all

How ${q://qid27/choicegroup/selectedchoices} do you feel your perceived level of seniority influences your experiences interacting with primary teams?

- Slightly
- Somewhat
- Very

The next questions are about your interactions with stewardship colleagues.

How influential do you feel your opinion is within your antimicrobial stewardship team?

- Not at all
- Slightly
- Somewhat
- Very

- Extremely

How do you feel your perceived gender identity influences your experiences interacting with members of your antimicrobial stewardship team?

- Negatively
- Positively
- Not at all

How ${q://qid31/choicegroup/selectedchoices} do you feel your perceived gender identity influences your experiences interacting with members of your antimicrobial stewardship team?

- Slightly
- Somewhat
- Very

How do you feel your perceived racial or ethnic identity influences your experiences interacting with members of your antimicrobial stewardship team?

- Negatively
- Positively
- Not at all

How ${q://qid34/choicegroup/selectedchoices} do you feel your perceived racial or ethnic identity influence your experiences interacting with members of your antimicrobial stewardship team?

- ◯ Slightly
- ◯ Somewhat
- ◯ Very

How do you feel your perceived level of seniority influences your experiences interacting with members of your antimicrobial stewardship team?

- ◯ Negatively
- ◯ Positively
- ◯ Not at all

How ${q://qid37/choicegroup/selectedchoices} do you feel your perceived level of seniority influences your experiences interacting with members of your antimicrobial stewardship team?

- ◯ Slightly
- ◯ Somewhat
- ◯ Very

${e://Field/Q1yes_Q31txt} ${e://Field/Q1no_Q31txt}

In your stewardship role, do you interact with health system representatives? This may include, but is not limited to, those in health system leadership (CMO, CEO, CFO).

- Yes
- No

How influential do you feel your opinion is within your health system?

- Not at all
- Slightly
- Somewhat
- Very
- Extremely

How do you feel your perceived gender identity influences your experiences interacting with health system representatives?

- Negatively
- Positively
- Not at all

How ${q://qid43/choicegroup/selectedchoices} do you feel your perceived gender identity influences your experiences interacting with health system representatives?

- Slightly
- Somewhat
- Very

How do you feel your perceived racial or ethnic identity influences your experiences interacting with health system representatives?

- Negatively
- Positively
- Not at all

How ${q://qid46/choicegroup/selectedchoices} do you feel your perceived racial or ethnic identity influences your experiences interacting with health system representatives?

- Slightly
- Somewhat
- Very

How do you feel your perceived level of seniority influences your experiences interacting with health system representatives?

- ◯ Negatively
- ◯ Positively
- ◯ Not at all

How ${q://qid49/choicegroup/selectedchoices} do you feel your perceived level of seniority influences your experiences interacting with health system representatives?

- ◯ Slightly
- ◯ Somewhat
- ◯ Very

The next questions are about your overall effectiveness and satisfaction as an antimicrobial stewardship provider.

How effective do you feel as an antimicrobial stewardship provider?

- ◯ Not at all
- ◯ Slightly
- ◯ Somewhat
- ◯ Very
- ◯ Extremely

How <u>satisfied</u> are you in your job as an antimicrobial stewardship provider?

- ◯ Not at all
- ◯ Slightly
- ◯ Somewhat
- ◯ Very
- ◯ Extremely

How <u>much pride</u> do you have in your work as an antimicrobial stewardship provider?

- ◯ None
- ◯ A little
- ◯ Some
- ◯ Quite a bit
- ◯ A great deal

How <u>stressful</u> do you find your job as an antimicrobial stewardship provider?

- ◯ Not at all
- ◯ Slightly
- ◯ Somewhat
- ◯ Very

- Extremely

The next questions are about distress, bias, and discrimination.

How often is your professional role or title misidentified in your discussions with healthcare personnel?

- Never
- Rarely
- Sometimes
- Very often
- Extremely often

In your role as an antimicrobial stewardship provider, have you experienced any bias or discrimination?

- Yes
- No

How often do you perceive bias against you during discussions with healthcare personnel?

- Never
- Rarely

- Sometimes
- Very often
- Extremely often

In the last year, how often have you avoided conversations with primary teams that you anticipated might be challenging?

- Never
- Rarely
- Sometimes
- Very often
- Extremely often

In the last year, how much anxiety have you felt when conducting stewardship activities that required direct communication with primary teams?

- None
- A little
- Some
- Quite a bit
- A great deal

In the last year, how often have you had recurrent positive thoughts about an interaction with healthcare personnel while preforming your duties as an antimicrobial stewardship provider?

- Never
- Rarely
- Sometimes
- Very often
- Extremely often

In the last year, how often have you had recurrent negative thoughts about an interaction with healthcare personnel while preforming your duties as an antimicrobial stewardship provider?

- Never
- Rarely
- Sometimes
- Very often
- Extremely often

While executing your duties as an antimicrobial stewardship provider in the last year, how often have you been…

	Never	Rarely	Sometimes	Very often	Extremely often
…yelled at?	○	○	○	○	○

	Never	Rarely	Sometimes	Very often	Extremely often
…verbally abused?	○	○	○	○	○
…belittled?	○	○	○	○	○

How often has your experience with bias or discrimination led you to question whether you want to continue to work as an antimicrobial stewardship provider?

- ○ Never
- ○ Rarely
- ○ Sometimes
- ○ Very often
- ○ Extremely often

During your training to become an antimicrobial stewardship provider, did you receive any mentorship?

- ○ Yes
- ○ No

How effective was the mentorship you received during your training to become an antimicrobial stewardship provider?

- Not at all
- Slightly
- Somewhat
- Very
- Extremely

During your training, did you have stewardship mentors of your same…

	Yes	No
…professional training?	○	○
…perceived gender identity?	○	○
…perceived racial or ethnic identity?	○	○

As an antimicrobial stewardship provider, do you receive ongoing mentorship?

- Yes
- No

How effective is the ongoing mentorship you receive as an antimicrobial stewardship provider?

- Not at all
- Slightly
- Somewhat
- Very
- Extremely

Do you have current stewardship mentors of your same…

	Yes	No
…professional training?	○	○
…perceived gender identity?	○	○
…perceived racial or ethnic identity?	○	○

Finally, we have some questions about you.

What is your profession?

- Pharmacist
- Physician
- Nurse
- Advance practice provider, such as physician assistant or nurse practitioner
- Pharmacy resident

○ Infectious Disease fellow
○ [ ] Other profession, please tell us:

What region do you practice in?

○ Northeast
○ Southeast
○ Mid-West
○ Southwest
○ West
○ Northwest

What is your age?

[ ]

How many years have you been practicing at your current level of training?

☐ [ ] Years
☐ Or, less than a year

How many years have you been practicing antimicrobial stewardship at your current program?

- [ ] ____ Years
- [ ] Or, less than a year

What is your gender?

____

Check all of the following that describe your race or ethnicity:

- [ ] American Indian or Alaskan Native
- [ ] Asian
- [ ] Black or African American
- [ ] Hispanic or Latino
- [ ] Middle Eastern or North African
- [ ] Native Hawaiian or Other Pacific Islander
- [ ] White
- [ ] ____ Other, please tell us:

Do you speak any languages other than English?

- ◯ Yes
- ◯ No

How many languages other than English do you speak?

What language do you feel most comfortable conversing in?

Block 2

These are all the questions we have for you at this time.

After the entire survey has been completed and you are ready to submit your answers, please click on the 'SUBMIT' button below. Once you click 'SUBMIT' you will not be able to re-enter the survey, but a link will be provided to a separate survey where you may sign up for the lottery

drawing or volunteer for a follow up interview.

Thank you for participating in this important study!

Powered by Qualtrics
