# Supplementary material for: Bias and discrimination perceived by antimicrobial stewards: a mixed-methods study
Source: Infect Control Hosp Epidemiol. 2025 Aug 19;46(9):910–9. doi: 10.1017/ice.2025.10224 (PMC12616225; doi:10.1017/ice.2025.10224)
Supplement: Tischendorf et al. supplementary material 3 — Tischendorf et al. supplementary material [file S0899823X25102249sup003.zip › Sup/Appendix C Complete Survey Responses.docx]

Supplemental Table 1. Survey results of all responses

| **Question text** | **Response description** | **All respondents (n=204)*** |
| --- | --- | --- |
| In situations requiring direct communication with primary teams, either verbal, text or electronic, in the last year, how often were your antimicrobial stewardship recommendations challenged? | (1) Never, (2) Rarely, (3) Sometimes, (4), Most of the time, (5) Always | 3.54 (1.26) |
| Overall, when interacting with primary teams, in the last year, how often were your recommendations as an antimicrobial stewardship provider ultimately enacted? | (1) Never, (2) Rarely, (3) Sometimes, (4), Most of the time, (5) Always | 3.76 (0.51) |
| Compared to other stewardship providers at your institution, in the last year how often were your recommendations challenged? | (1) A great deal less often, (2) Somewhat less often, (3) Slightly less often, (4) About as often, (5) Slightly more often, (6) Somewhat more often, (7) A great deal more often | 6.7 (0.76) |
| While conducting your duties as an antimicrobial stewardship provider, how do you feel your perceived gender identity influences your experiences interacting with primary teams? | (1) Very negatively, (2) Somewhat negatively, (3) Slightly negatively, (4) Not at all, (5) Slightly positively, (6) Somewhat positively, (7) Very positively | 4.07 (1.15) |
| While conducting your duties as an antimicrobial stewardship provider, how do you feel your perceived racial or ethnic identity influences your experiences interacting with primary teams? | (1) Very negatively, (2) Somewhat negatively, (3) Slightly negatively, (4) Not at all, (5) Slightly positively, (6) Somewhat positively, (7) Very positively | 4.16 (0.83) |
| While conducting your duties as an antimicrobial stewardship provider, how do you feel your perceived seniority influences your experiences interacting with primary teams? | (1) Very negatively, (2) Somewhat negatively, (3) Slightly negatively, (4) Not at all, (5) Slightly positively, (6) Somewhat positively, (7) Very positively | 4.64 (1.77) |
| How influential do you feel your opinion is within your antimicrobial stewardship team? | (1) Not at all, (2) Slightly, (3) Somewhat, (4) Very, (5) Extremely | 3.71 (0.83) |
| How do you feel your perceived gender identity influences your experiences interacting with members of your antimicrobial stewardship team? | (1) Very negatively, (2) Somewhat negatively, (3) Slightly negatively, (4) Not at all, (5) Slightly positively, (6) Somewhat positively, (7) Very positively | 4.18 (0.92) |
| How do you feel your perceived racial or ethnic identity influences your experiences interacting with members of your antimicrobial stewardship team? | (1) Very negatively, (2) Somewhat negatively, (3) Slightly negatively, (4) Not at all, (5) Slightly positively, (6) Somewhat positively, (7) Very positively | 4.31 (0.83) |
| How do you feel your perceived seniority influences your experiences interacting with members of your antimicrobial stewardship team? | (1) Very negatively, (2) Somewhat negatively, (3) Slightly negatively, (4) Not at all, (5) Slightly positively, (6) Somewhat positively, (7) Very positively | 4.73 (1.61) |
| In your stewardship role, do you interact with health system representatives? This may include, but is not limited to, those in health system leadership (CMO, CEO, CFO). | Binary | n=69, 34% |
| How influential do you feel your opinion is within your health system? | (1) Not at all, (2) Slightly, (3) Somewhat, (4) Very, (5) Extremely | 3.04 (0.9) |
| How do you feel your perceived gender identity influences your experiences interacting with health system representatives? | (1) Very negatively, (2) Somewhat negatively, (3) Slightly negatively, (4) Not at all, (5) Slightly positively, (6) Somewhat positively, (7) Very positively | 4.03 (1.29) |
| How do you feel your perceived racial or ethnic identity influences your experiences interacting with health system representatives? | (1) Very negatively, (2) Somewhat negatively, (3) Slightly negatively, (4) Not at all, (5) Slightly positively, (6) Somewhat positively, (7) Very positively | 4.36 (1.09) |
| How do you feel your perceived seniority influences your experiences interacting with health system representatives? | (1) Very negatively, (2) Somewhat negatively, (3) Slightly negatively, (4) Not at all, (5) Slightly positively, (6) Somewhat positively, (7) Very positively | 4.47 (1.69) |
| How effective do you feel as an antimicrobial stewardship provider? | (1) Not at all, (2) Slightly, (3) Somewhat, (4) Very, (5) Extremely | 3.39 (0.80) |
| How satisfied are you in your job as an antimicrobial stewardship provider? | (1) Not at all, (2) Slightly, (3) Somewhat, (4) Very, (5) Extremely | 3.44 (0.81) |
| How much pride do you have in your work as an antimicrobial stewardship provider? | (1) None, (2) A little, (3) Some, (4) Quite a bit, (5) A great deal | 4.03 (0.88) |
| How stressful do you find your job as an antimicrobial stewardship provider? | (1) Not at all, (2) Slightly, (3) Somewhat, (4) Very, (5) Extremely | 3.10 (0.89) |
| How often is your professional role or title misidentified in your discussions with healthcare personnel? | (1) Never, (2) Rarely, (3) Sometimes, (4), Most of the time, (5) Always | 2.62 (0.91) |
| In your role as an antimicrobial stewardship provider, have you experienced any bias or discrimination? | Binary | n=85, 41% |
| How often do you perceive bias against you during discussions with healthcare personnel? | (1) Never, (2) Rarely, (3) Sometimes, (4), Most of the time, (5) Always | 2.17 (0.82) |
| In the last year, how often have you avoided conversations with primary teams that you anticipated might be challenging? | (1) Never, (2) Rarely, (3) Sometimes, (4), Most of the time, (5) Always | 2.47 (0.83) |
| In the last year, how often have you had recurrent positive thoughts about an interaction with healthcare personnel while preforming your duties as an antimicrobial stewardship provider? | (1) Never, (2) Rarely, (3) Sometimes, (4), Most of the time, (5) Always | 3.17 (0.80) |
| In the last year, how often have you had recurrent negative thoughts about an interaction with healthcare personnel while preforming your duties as an antimicrobial stewardship provider? | (1) Never, (2) Rarely, (3) Sometimes, (4), Most of the time, (5) Always | 2.78 (0.83) |
| While executing your duties as an antimicrobial stewardship provider in the last year, how often have you been yelled at? | (1) Never, (2) Rarely, (3) Sometimes, (4), Most of the time, (5) Always | 1.64 (0.86) |
| While executing your duties as an antimicrobial stewardship provider in the last year, how often have you been verbally abused? | (1) Never, (2) Rarely, (3) Sometimes, (4), Most of the time, (5) Always | 1.48 (0.80) |
| While executing your duties as an antimicrobial stewardship provider in the last year, how often have you been belittled? | (1) Never, (2) Rarely, (3) Sometimes, (4), Most of the time, (5) Always | 2.05 (1.00) |
| How often has your experience with bias or discrimination led you to question whether you want to continue to work as an antimicrobial stewardship provider? | (1) Never, (2) Rarely, (3) Sometimes, (4), Most of the time, (5) Always | 2.44 (0.97) |
| During your training to become an antimicrobial stewardship provider, did you receive any mentorship? | Binary | n=141, 69% |
| How effective was the mentorship you received during your training to become an antimicrobial stewardship provider? | (1) Not at all, (2) Slightly, (3) Somewhat, (4) Very, (5) Extremely | 3.77 (0.94) |
| During your training, did you have stewardship mentors of your same professional training? | Binary | n=115 (56%) |
| During your training, did you have stewardship mentors of your same perceived gender identity? | Binary | n=91 (45%) |
| During your training, did you have stewardship mentors of your same racial or ethnic identity? | Binary | n=97 (48%) |
| As an antimicrobial stewardship provider, do you receive ongoing mentorship? | Binary | n=82 (40%) |
| How effective is the ongoing mentorship you receive as an antimicrobial stewardship provider? | (1) Not at all, (2) Slightly, (3) Somewhat, (4) Very, (5) Extremely | 3.59 (0.92) |
| Do you have current stewardship mentors of your same professional training? | Binary | N=62 (30%) |
| Do you have current stewardship mentors of your same perceived gender identity? | Binary | n=58 (28%) |
| Do you have current stewardship mentors of you same perceived racial or ethnic identity? | Binary | n=61 (30%) |

*Mean and standard deviations are shared for 5-point and 7-point Likert scale responses; number and percent of respondents indicating “yes” are shared for binary (yes/no) responses.
